# Supplementary material for: The inorganic pyrophosphatases of microorganisms: a structural and functional review
Source: PeerJ. 2024 Jun 24;12:e17496. doi: 10.7717/peerj.17496 (PMC11210485; doi:10.7717/peerj.17496)

Supplementary Figure 1. *Clostridium tetani* encodes for both a family II CBS-domain enzyme and a m-PPase. Panel A, UniProt entry A0A4Q0VED8 corresponds to a family II PPase; the model is shown in rainbow color. Panel B compares the C. tetani enzyme with the B. subtilis family II PPase (RMSD=2.43, TM-score 0.462). Panel C is the model for the predicted K+ stimulated m-PPase of C. tetani (UniProt A0A4Q0V5P6), rainbow color scheme.

Supplementary Figure 2. *S. carlsbergensis* (*Saccharomyces pastorianus*) cytoplasmic PPase is inconsistent with a membrane-bound enzyme. UniProt entry A0A6C1DXI6 encodes for a second PPase (the family I enzyme consistent with other family I enzymes is A0A6C1DM74, and an almost identical copy, UniProt A0A6C1E2Z3). The model shows that the N-terminal domain is also a helix (as for E. histolytica) with extensive negative charge. This suggests that the enzyme may not be associated with the membrane but co-purified along with a cellular membrane.

Supplementary Figure 3. Protter analysis of the selected m-PPases is shown in Figures 10 and 12. The number indicates the position of each cluster of enzymes in Figure 10. Asterix indicates the Protter prediction for *R. rubrum* enzyme, and the model suggests the position of the catalytic residues (in red). The upper part of each figure corresponds to the outside of the membrane, and the lower part to the cytoplasmic side.

Supplementary Figure 4. The finding of an OmpA-like domain in a m-PPase. In search of the diversity of m-PPases and group I of Figure 10, we found C-terminal extensions in the examples analyzed; by searching in UniProt by protein length, we found that in Paucibacter KCTC 42545, the m-PPase (UniProt A0A0U2VTE8) was 810. In panel A, the AlphaFold2 model shows that the C-terminal end contains an extra domain. In panel B, HMMER analysis showed that the extra domain exhibits similarity to OmpA protein, and the sequence is conserved among bacteria, as examples: *Burkholderiales bacterium, Rubrivirax sp., Rhizobacter, Acidovorax sp., Polaromonas sp*. The domain shows variable conservation in the extension of the sequence (upper graph), and the distribution is shown to be in bacteria exclusively (middle and lower images). In Panel C, a comparison of the full-length model of Escherichia coli K-12 OmpA (UniProt P0A910) is presented in a rainbow color scheme. Also, the structural alignment shown in Panel D (RMSD=5.88, TM-score= 0.052), although very low, suggests that the extra domain in the m-PPase (in blue) is a remote resemblance to OmpA (in red).

**Supplementary Figure 1**

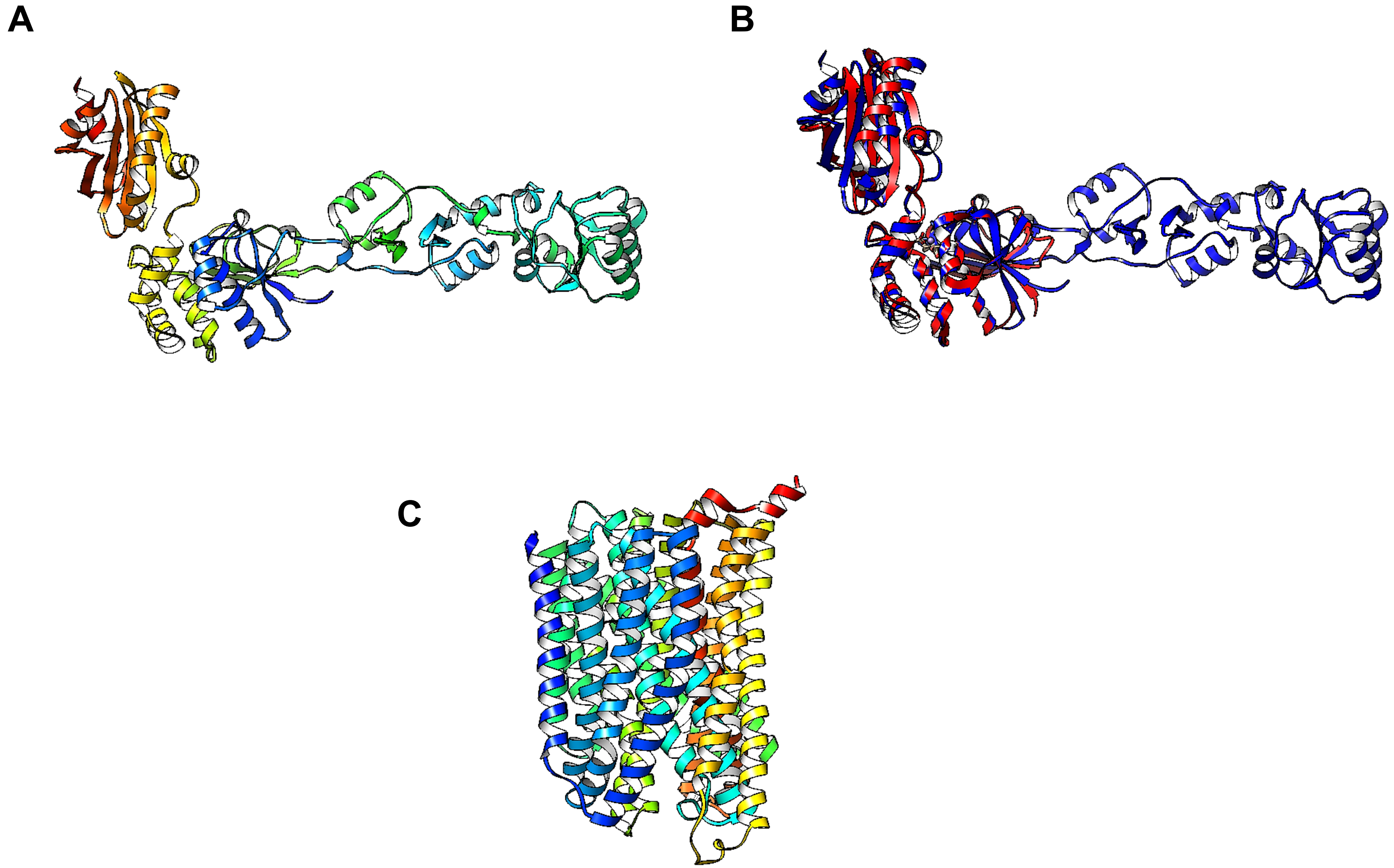

# Supplementary Figure 2

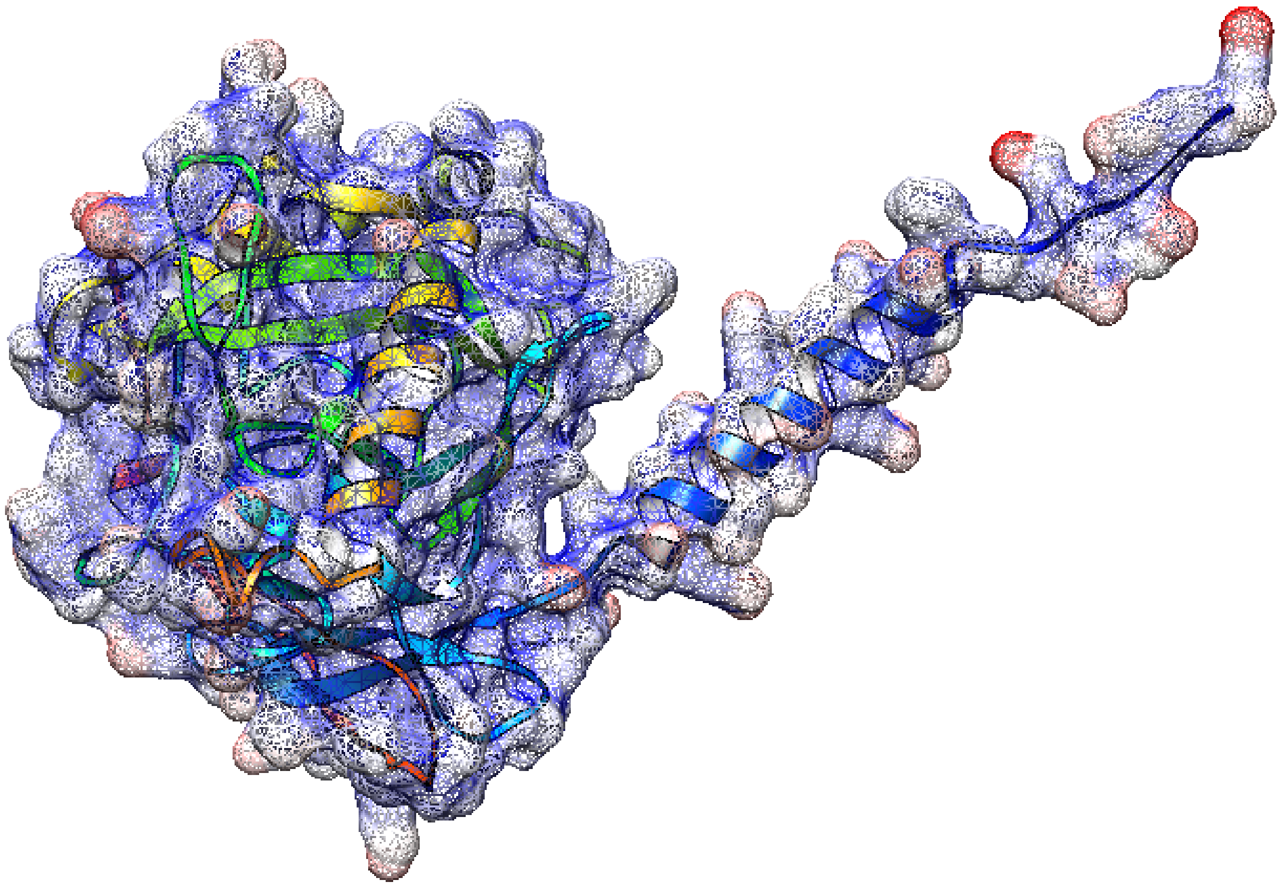

**Supplementary Figure 3**

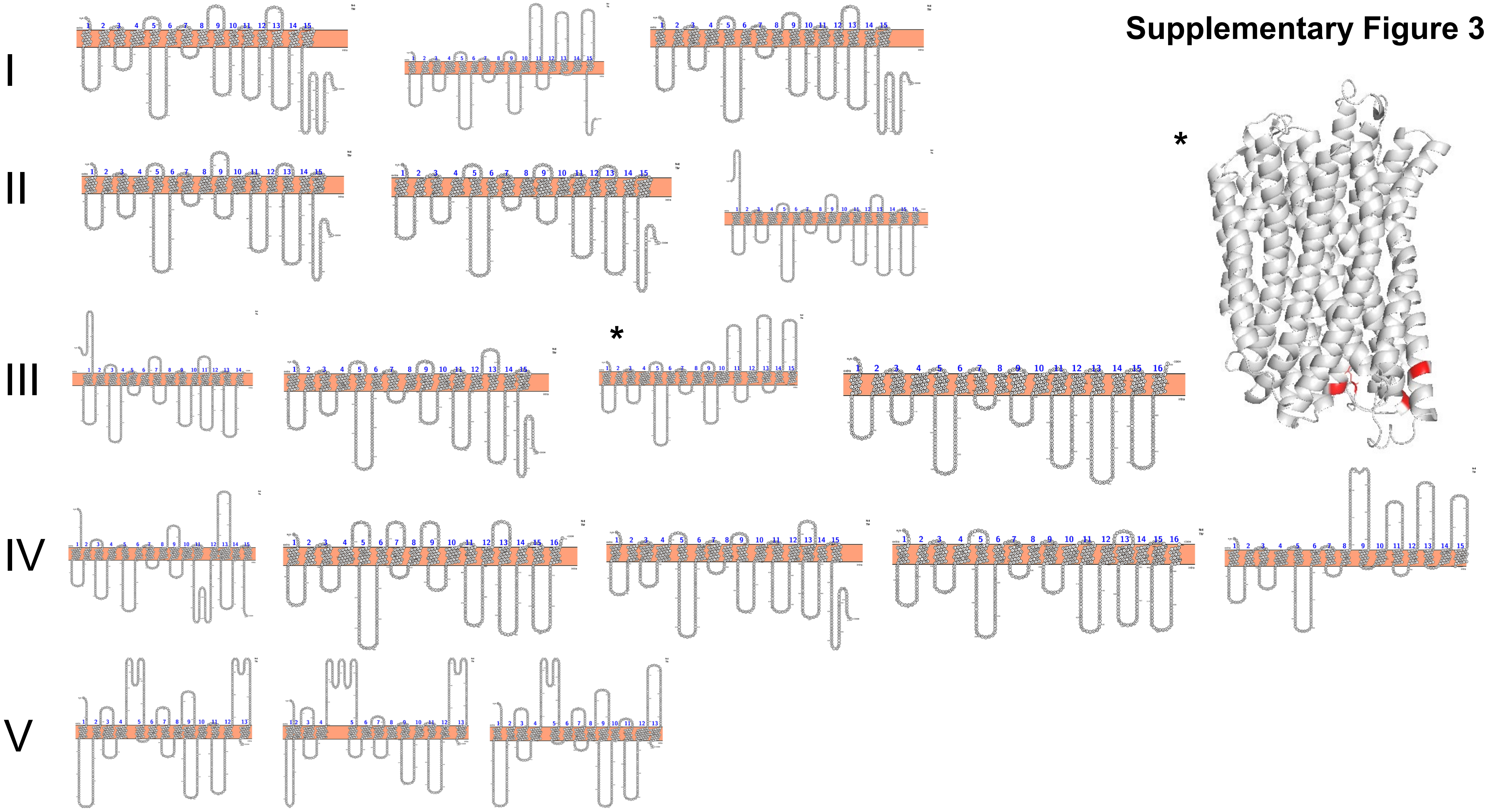

# Supplementary Figure 4

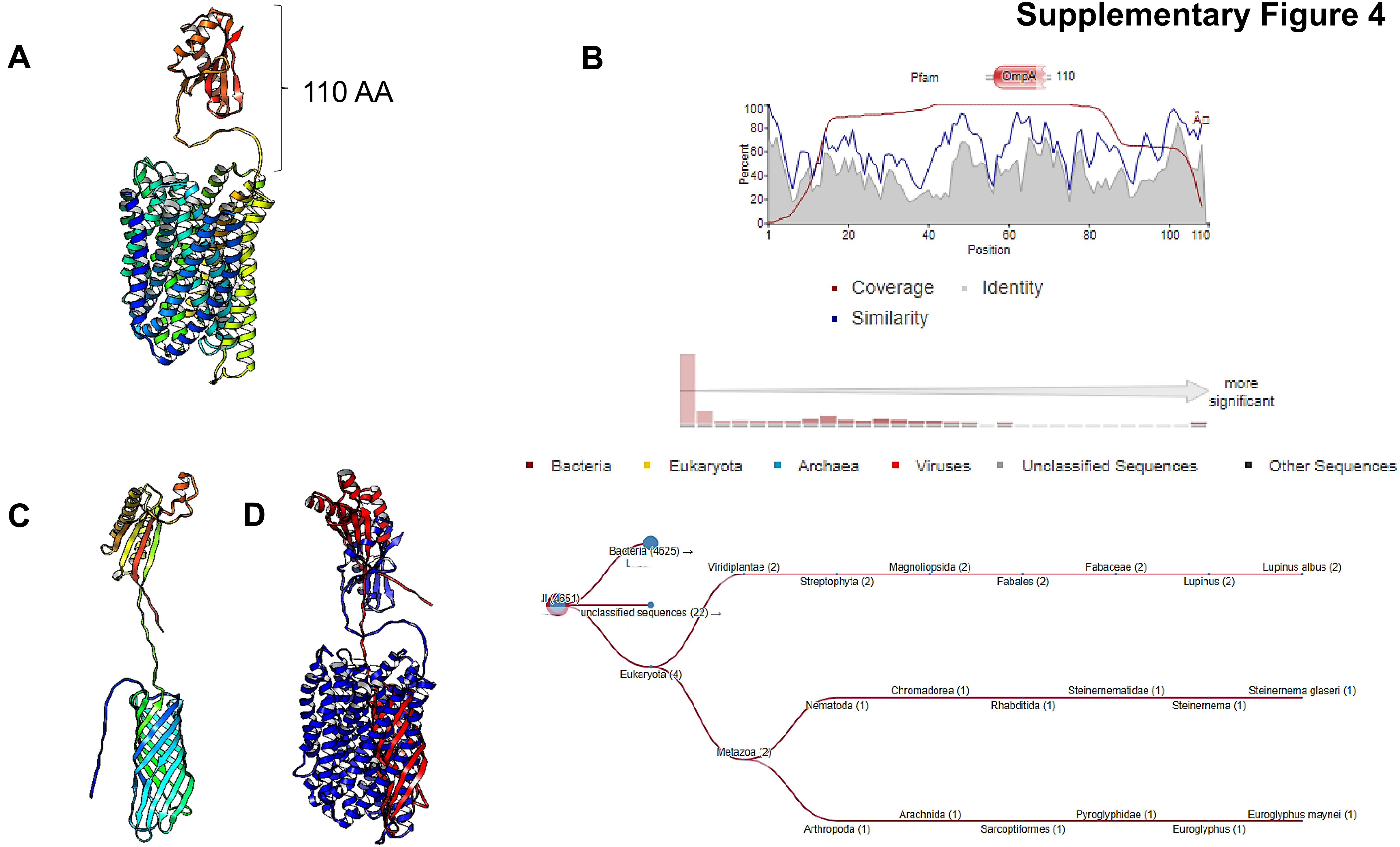

Supplement: Supplemental Information 2 — Supplementary Figure 1. Clostridium tetani encodes for both a family II CBS-domain enzyme and a m-PPase. (A), UniProt entry A0A4Q0VED8 corresponds to a family II PPase; the model is shown in rainbow color. (B) compares the C. tetani enzyme with the B. subtilis family II PPase (RMSD = 2.43, TM-score 0.462). (C) is the model for the predicted K+ stimulated m-PPase of C. tetani (UniProt A0A4Q0V5P6), rainbow color scheme. Supplementary Figure 2. S. carlsbergensis (Saccharomyces pastorianus) cytoplasmic PPase is inconsistent with a membrane-bound enzyme. UniProt entry A0A6C1DXI6 encodes for a second PPase (the family I enzyme consistent with other family I enzymes is A0A6C1DM74, and an almost identical copy, UniProt A0A6C1E2Z3). The model shows that the N-terminal domain is also a helix (as for E. histolytica) with extensive negative charge. This suggests that the enzyme may not be associated with the membrane but co-purified along with a cellular membrane. Supplementary Figure 3. Protter analysis of the selected m-PPases is shown in Figs. 10 and 12. The number indicates the position of each cluster of enzymes in Fig. 10. Asterix indicates the Protter prediction for R. rubrum enzyme, and the model suggests the position of the catalytic residues (in red). The upper part of each figure corresponds to the outside of the membrane, and the lower part to the cytoplasmic side. Supplementary Figure 4. The finding of an OmpA-like domain in a m-PPase. In search of the diversity of m-PPases and group I of Fig. 10, we found C-terminal extensions in the examples analyzed; by searching in UniProt by protein length, we found that in Paucibacter KCTC 42545, the m-PPase (UniProt A0A0U2VTE8) was 810. In (A), the AlphaFold2 model shows that the C-terminal end contains an extra domain. In (B), HMMER analysis showed that the extra domain exhibits similarity to OmpA protein, and the sequence is conserved among bacteria, as examples: Burkholderiales bacterium, Rubrivirax sp., Rhizobacter, Acid [file peerj-12-17496-s002.pdf]
